# Case report: Mutation in *NPPA* gene as a cause of fibrotic atrial myopathy

**DOI:** 10.3389/fcvm.2023.1149717

**Published:** 2023-06-08

**Authors:** Pedro Silva Cunha, Diana Oliveira Antunes, Sérgio Laranjo, Ana Coutinho, João Abecasis, Mário Martins Oliveira

**Affiliations:** ^1^ Arrhythmology, Pacing and Electrophysiology Unit, Cardiology Service, Santa Marta Hospital, Centro Hospitalar Universitário Lisboa Central, Lisbon, Portugal; ^2^ Faculdade de Medicina, Universidade de Lisboa, Lisbon, Portugal; ^3^ Cardiovascular Department, Hospital Lusíadas Lisboa, Lisbon, Portugal; ^4^ Genetics Department, Hospital Dona Estefânia, Centro Hospitalar Universitário Lisboa Central, Lisbon, Portugal; ^5^ GenoMed Diagnóstico Medicina Molecular, Instituto de Medicina Molecular, Lisbon, Portugal; ^6^ NOVA Medical School, Universidade NOVA de Lisboa, Lisbon, Portugal

**Keywords:** atrial fibrillation, *NPPA* gene, mutation—genetics, fibrosis, atrial myopathy

## Abstract

Early-onset atrial fibrillation (AF) can be the manifestation of a genetic atrial myopathy. However, specific genetic identification of a mutation causing atrial fibrosis is rare. We report a case of a young patient with an asymptomatic AF, diagnosed during a routine examination. The cardiac MRI revealed extensive atrial fibrosis and the electrophysiology study showed extensive areas of low voltage. The genetic investigation identified a homozygous pathogenic variant in the *NPPA* gene in the index case and the presence of the variant in heterozygosity in both parents.

## Introduction

Atrial fibrillation (AF) is a complex disease where several environmental and genetic risk factors contribute to its genesis. In recent years, rapid progress has been made in identifying the genetic basis for this highly prevalent arrhythmia.

Early-onset AF can be the manifestation of an atrial myopathy. However, specific genetic identification of a mutation causing atrial fibrosis is rare.

Here, we report a case of a young patient with atrial fibrillation and absence of previously known cardiovascular disease or cardiovascular risk factors, in which we identified a homozygous pathogenic variant in *NPPA* gene (by targeted gene panel for inherited cardiac diseases from whole-exome sequencing) and a phenotype characterised by persistent atrial fibrillation and bi-atrial extensive areas of low voltage (generalised fibrosis). This case report is exemplary of a genetic arrhythmogenic atrial cardiomyopathy.

## Case report

A 31-year-old man, asymptomatic with an unremarkable medical history and regularly observed by occupational medicine, was diagnosed with atrial fibrillation (of unknown duration since a previous ECG performed 24 months before presented normal sinus rhythm) during his routine health check. He was subsequently referred to our arrhythmia department for evaluation.

**Figure 1:**
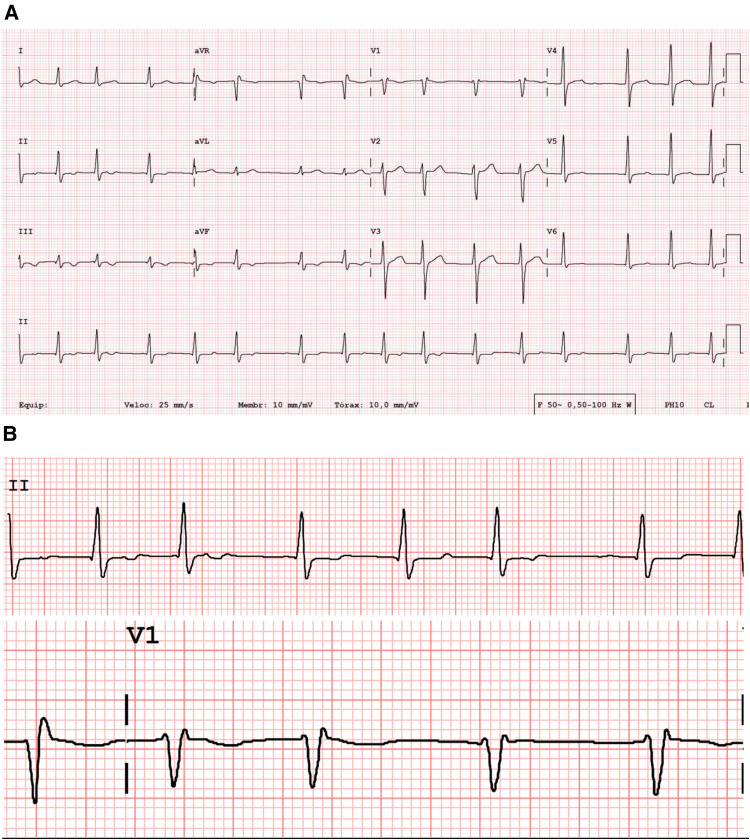
Standard 12-lead surface ECG (**A**), with a very low voltage atrial activity and irregularly irregular (with no pattern to the RR intervals) ventricular response with a rate of 92 beats/mi. QRS width of 100 msec; QTc 463 msec. Magnification of leads DII and V1 (**B**), showing almost undetectable atrial electrical activity.

The patient had no history of structural heart disease or tobacco or alcohol use. Except for irregular pulse, the physical findings were within the normal range on the baseline examination. The surface ECG presented atrial fibrillation—with a very low voltage (Figure 1) -and the transthoracic echocardiography estimated the LV ejection fraction to be 65%. The left atrial dimension was 35 ml/m2.

The patient was started on oral flecainide (weight-adjusted), bisoprolol, and non-vitamin K oral anticoagulant, and four weeks later, was submitted to electrical cardioversion with success. However, one week after cardioversion, the patient resumed atrial fibrillation. The medical team discussed with the patient the state of the illness and the possible benefits and risks of various treatments. Pulmonary vein isolation was addressed in the face of the results of the EAST-AFNET 4 trial (1) that indicate a rhythm-control strategy is superior to usual care (rate control in the majority of cases) in improving CV outcomes at five years and also the results of the author's centre that reported that a single ablation procedure in patients with persistent AF, resulted in 62.2% freedom from AF at a 22-month follow-up (2). Therefore, the option for catheter ablation treatment was chosen.

**Figure 2:**
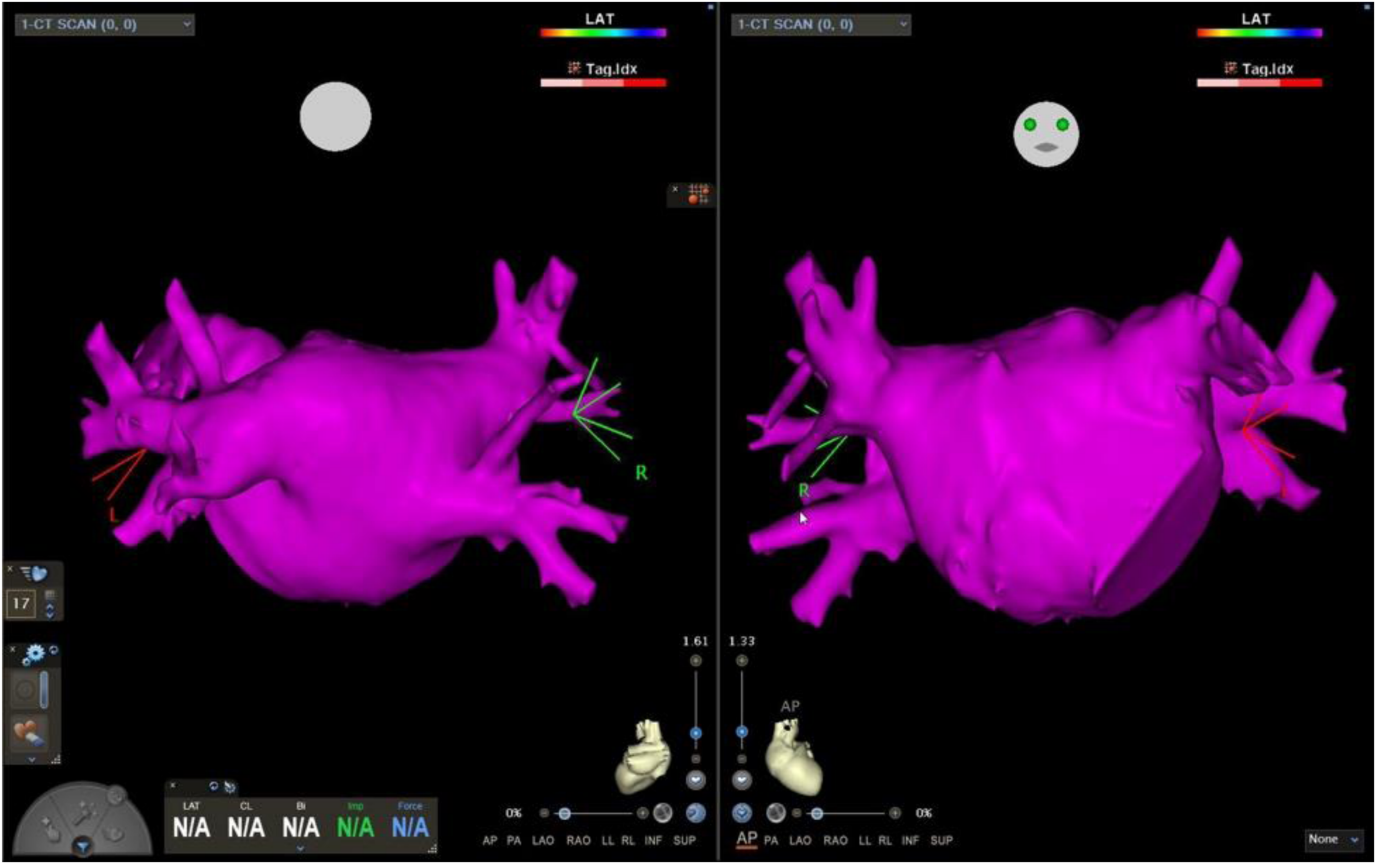
Left atrial CT scan segmentation. Cardiac computed tomography, with a left atrial volume of 70 ml and a left pulmonary venous drainage pattern R2a/L1.

Cardiac computed tomography (CT) evaluation before the electrophysiological study revealed no significant stenotic lesions in the coronary arteries and no structural heart disease, with a left atrial volume of 70 ml and a left pulmonary venous drainage pattern R2a/L1 (Figure 2) (3).

### Ablation procedure

**Figure 3:**
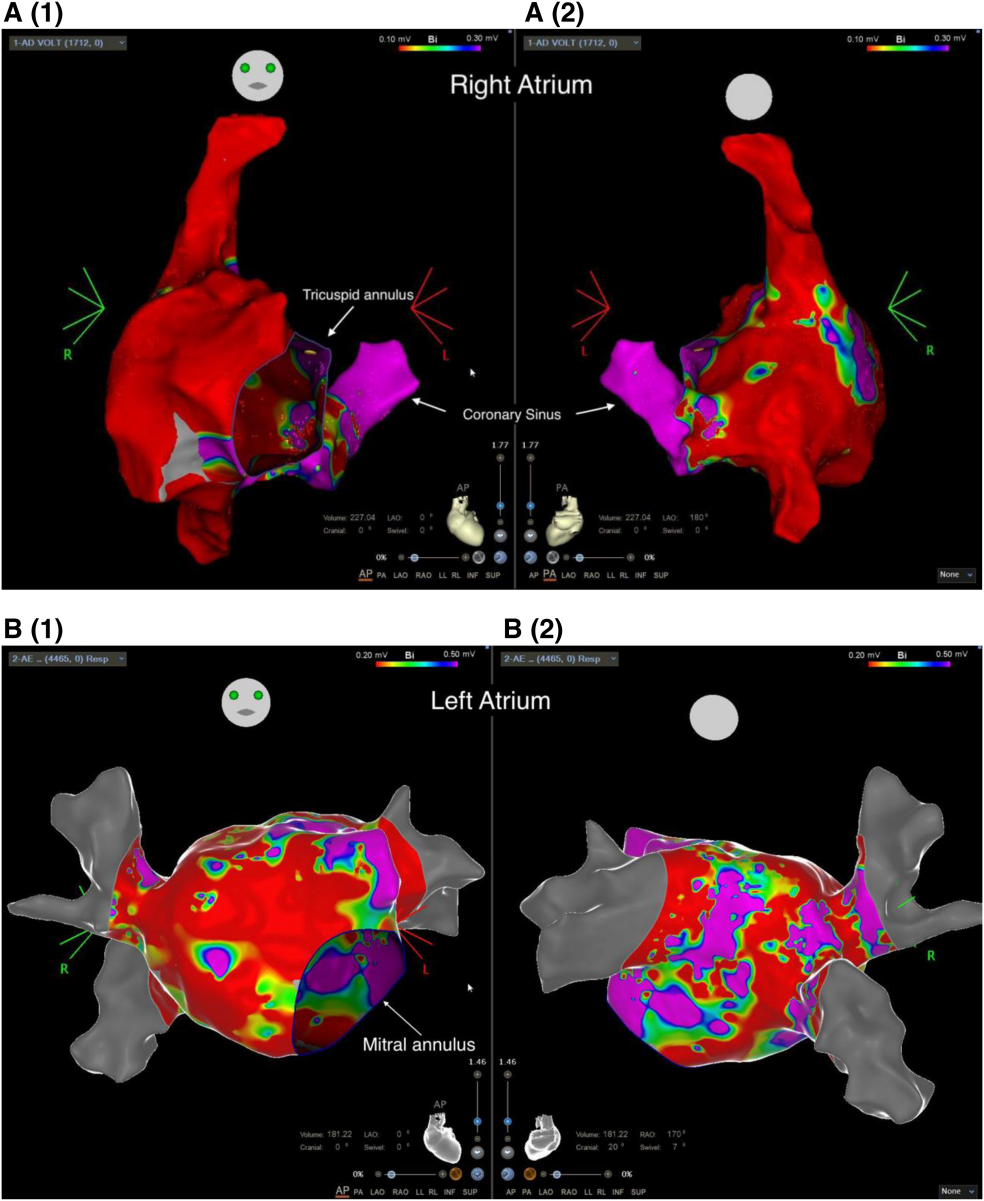
Voltage map of the right (**A**) and the left atrium (**B**), depicting extensive areas of low voltage (red) (cut-off 0.20−0.50 mV). (**A**1 and **B**1) Antero-Posterior view; (**A**2 and **B**2) posteroanterior view.

A three-dimensional (3D) mapping system (Carto, Biosense Webster, Diamond Bar, California) was used for electro-anatomical mapping and ablation. First, a transseptal puncture from the right atrium to the LA was completed under fluoroscopic guidance. Next, the patient underwent high-density mapping of bi-atrial voltage using the PentaRay multipolar catheter (Biosense Webster, Inc). In the mapping system, the cut-off values for defining low-voltage areas (LVAs) were <0.5 mV for low voltage, <0.2 mV for dense scar, and >0.5 mV for normal voltage. The voltage mapping points were obtained in atrial fibrillation before the pulmonary veins’ ablation. Here, the number of points was >1,500. The right and left atrial maps revealed extensive LVAs (Figure 3). Wide antral pulmonary vein isolation was performed, and the patient was cardioverted at the end of the procedure. Twelve hours after the PVI and cardioversion, the AF returned. In the face of the extensive LVA, the medical team opted not to convert the arrhythmia.

Considering the clinical context of AF at a young age and extensive LVA in invasive 3D mapping, we decided to perform a genetic study.

### Genetic study and results

**Figure 4:**
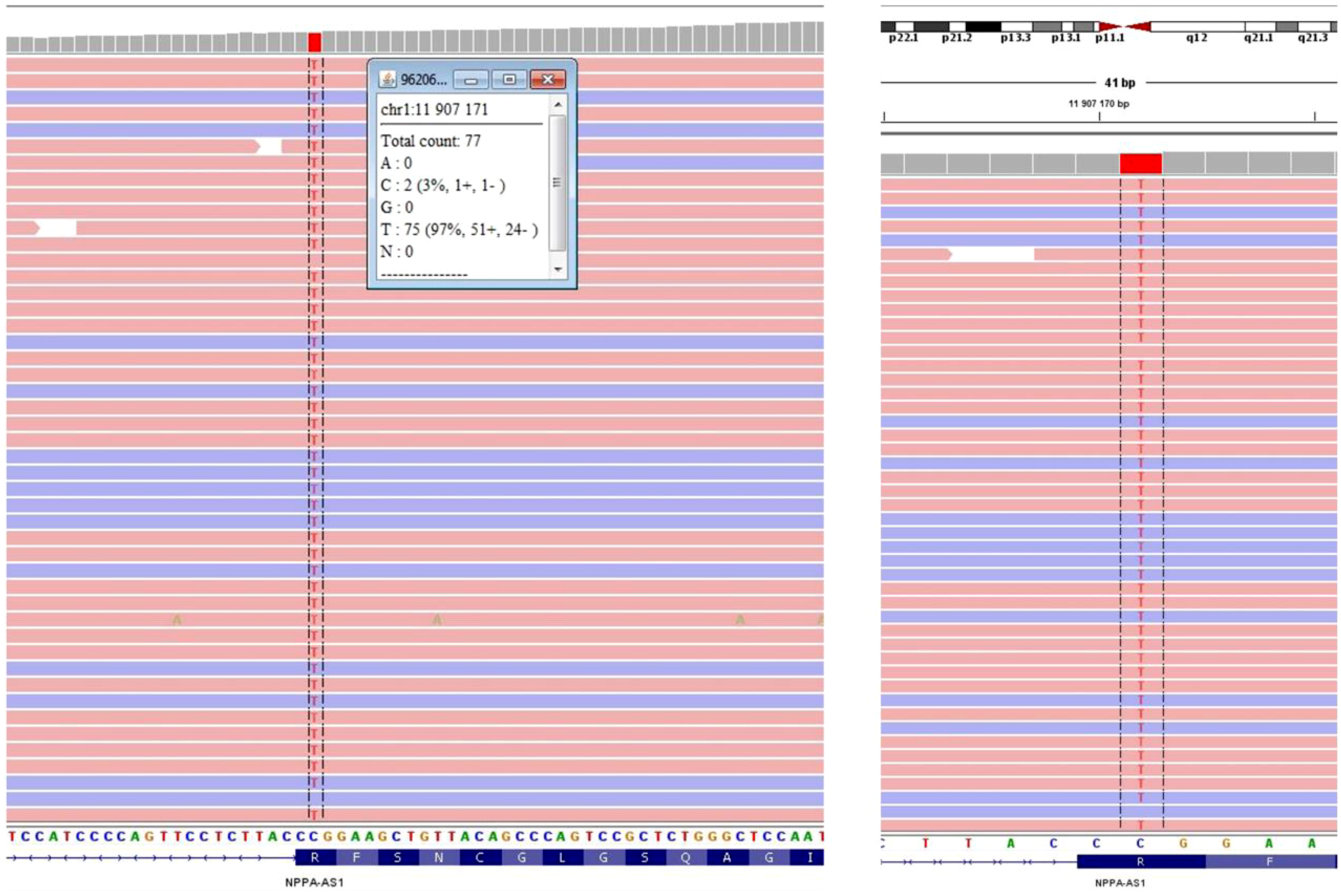
Visualization of next-generation sequencing results, showing the variant c.449G > A, p.(Arg150Gln) in the NPPA gene as homozygous in the index case.

DNA was obtained from the peripheral blood of the patient. Whole exome sequencing was performed through Next-generation sequencing with Twist human core exome plus RefSeq extension (Twist Bioscience) in Illumina NovaSeq 6,000 platform. A targeted panel for the inherited cardiac disease were analysed (in Supplementary Material), which revealed the variant c.449G > A, p.(Arg150Gln) in homozygosity in NPPA gene (Figure 4) classified as pathogenic, according to guidelines ACMG/ACGS 2020 (P—PM3_vstr, PP1_str).

**Figure 5:**
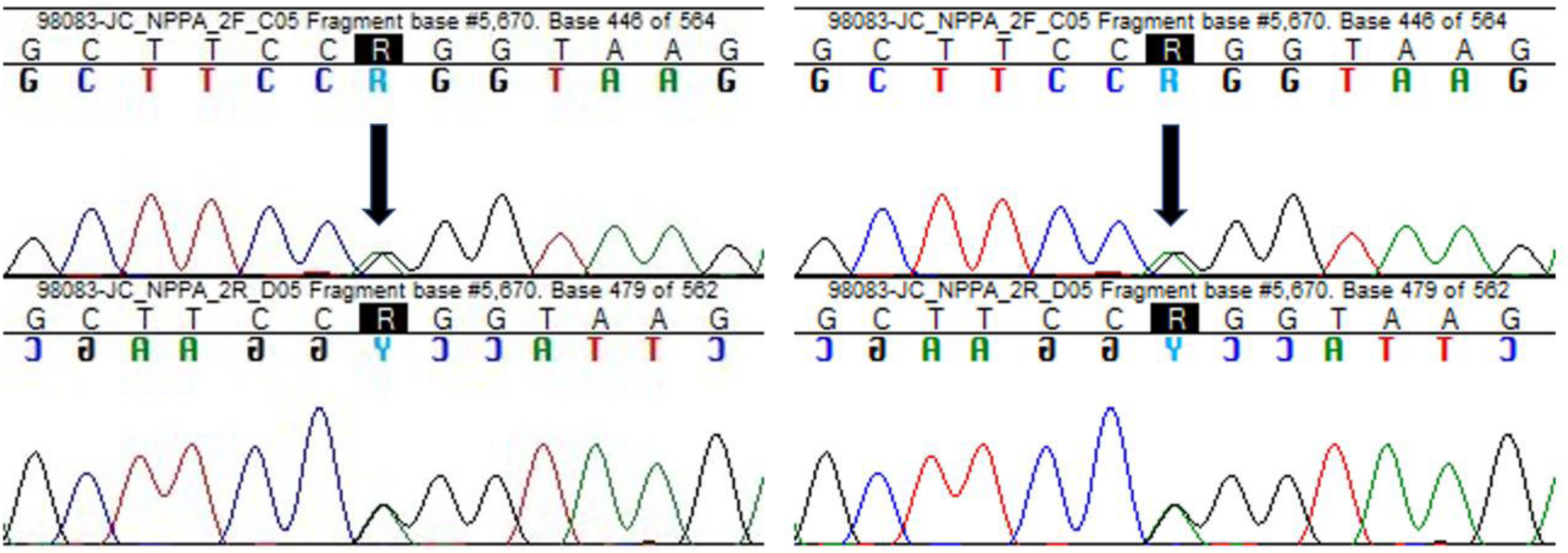
Sanger sequencing showing the variant c.449G > A, p.(Arg150Gln) in NPPA gene, identified in heterozygosity both in the father and the mother of the patient. Black arrows indicate the point mutation.

Familiar segregation studies reveal the consanguinity of the parents, and the presence of the variant in heterozygosity (Figure 5) in both parents was confirmed. Furthermore, the asymptomatic brother was also proved to be a carrier of the variant in heterozygosity.

### Follow-up

**Figure 6:**
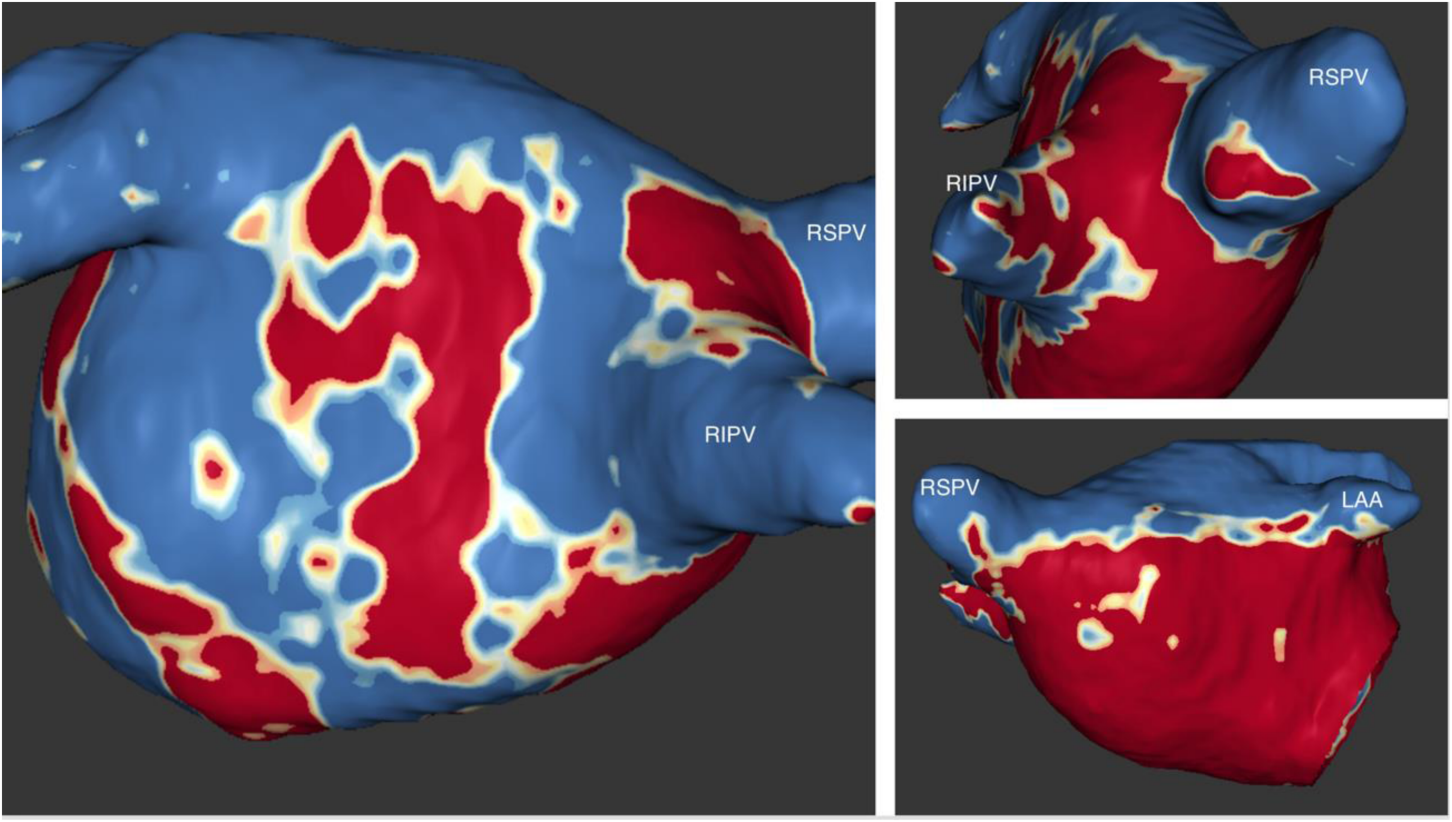
Images from late gadolinium enhancement (LGE) MRI. Fibrosis assessment with the ADAS 3 D software. The three-dimensional LA model from MRI shows extensive areas of fibrosis (dense scar) involving the posterior wall and the anterior and septal walls. LAA, left atrial appendage; RSVP, right superior pulmonary vein; RIPV, right inferior pulmonary vein.

Eight months after the ablation procedure, the patient remained asymptomatic without physical activity limitation and under oral anticoagulation and bisoprolol. The rhythm recorded on the routine Holter was atrial fibrillation, confirmed by a surface electrocardiogram. In addition, cardiac magnetic resonance imaging was performed (Figure 6), where extensive areas of fibrosis could be identified. Eighteen months after the ablation procedure, according to the European Heart Rhythm Association (EHRA) score of AF-related symptoms, the patient classifies as score I (4, 5). Integrating the clinical context and the results of the cardiac MRI, the patient and physician accepted the presence of AF, and no further attempts to restore/maintain sinus rhythm were undertaken.

## Discussion

Atrial natriuretic peptides (ANP) establish a relationship between the heart and the kidneys. ANP is a powerful hormone with natriuretic, diuretic, and hypotensive actions (6). Its secretion occurs in the right atrium as a reaction to atrial stretching due to factors like hypervolemia or hypertension. Furthermore, this hormone regulates sodium homeostasis (7), vascular remodelling, and energy metabolism (8). In addition to the functions mentioned above, ANP and B-type natriuretic peptide (BNP) have also been shown to exert antifibrotic and antihypertrophic effects within the heart (8, 9).

ANP is encoded by the *NPPA* gene (10) located on chromosome 1 in the human genome and is primarily expressed by atrial myocytes. ANP derives from its precursors pre-pro-ANP and pro-ANP (11).

The *NPPA* gene encodes a 151-amino acid polypeptide known as preproANP. A post-translational modification process cleaves the 25 amino acid signal sequence to produce proANP, a 126 amino acid peptide stored in intracellular granules of atrial myocytes (12).

In 2008 Hodgson-Zingman et al. (13) first identified a truncating frameshift mutation in *NPPA* in a family with an autosomal dominant inheritance pattern of AF. This specific mutation caused a two-base pair deletion in exon three that eliminated the original stop codon, giving origin to 12 new amino acids to be appended to the C terminus of the mature peptide. Later, Disertori et al. (14) identified by linkage analysis a locus at 1p36.22 that contained the Natriuretic Peptide Precursor A gene and, by sequencing, identified the homozygous missense mutation (p.Arg150Gln). The same authors (14, 15) described a population of patients in whom the following clinical characteristics were observed: clinical onset in adulthood; biatrial dilatation (up to giant size); early supraventricular arrhythmias with progressive loss of atrial electric activity to an atrial standstill; thromboembolic complications; and during the long-term course of the disease a stable, normal left ventricular function.

A population-based association study in China (16) with a case-control design supported that variants in *NPPA* confer the risk of lone AF. These results establish the association between a common variant (a heterozygous variant p.Ile138Thr) in *NPPA* and lone AF. Several other variants in *NPPA*, including p.Ser64Arg, p.Gln93Glu, and p.Ala117Val, were later also linked to AF (17, 18).

A study that comprehensively examined the functional consequences of the frameshift mutation of ANP (19) found data that indicate that the familial ANP mutation associated with atrial fibrillation has only minor effects on natriuretic peptide receptor interactions but markedly modifies peptide proteolysis. The authors conclude that this mutation increased the resistance of ANP to degradation, in essence causing an increase in ANP-mediated signalling. ANP exerts its effects by increasing the amounts of cyclic guanosine monophosphate (cGMP) circulating in target tissues (20).

To further study the biological implications, a group of researchers have conducted studies in mice that knocked out either the gene for ANP or the gene for natriuretic peptide receptor-A (NPR-A). Cheng et al. (21) demonstrated that AF-associated human variant p.Ile138Thr in natriuretic peptide A (*NPPA*) encoding the atrial natriuretic peptide (ANP) causes inflammation, fibroblast activation, atrial fibrosis, and AF in knock-in (KI) rats. This variant inhibits the interaction between ANP and its receptor and reduces intracellular cGMP levels. Although the exact molecular mechanisms are still unclear, this study observed that mutant ANP activates multiple innate immunity pathways, including TNF-α, NF-κB, and IL-1β signalling. In addition, the mutant ANP induces cardiac fibroblast (CFs) differentiation to myofibroblasts and promotes CF proliferation and fibrosis. These results suggest that *NPPA* variant p.Ile138Thr causes AF by starting innate immunity by inflammasome activation (22).

In the above-described clinical case, the knowledge of the genetic variant is essential, leading us to make therapeutic options based on similar variants reported in the literature. For instance, even though the patient had a CHADS-VASC risk score of 0, it was decided to maintain life-long oral anticoagulation given the information from the study by Disertori et al. (14), in which 13 members of a family were followed for 37 years, and one of the complications reported was the occurrence of thromboembolic events.

Our clinical case is unique since it has extensive documentation of atrial myopathy through invasive intracavitary tissue voltage assessment (endocavitary mapping with the 3D CARTO system) and the evaluation by magnetic resonance imaging displaying fibrotic areas. The recognition of extensive atrial fibrosis represents essential data in the clinical decision since the dependence of marked disease on the substrate is apparent as the cause of atrial fibrillation. Therefore, the repetition of the catheter ablation would be superfluous in the approach of this specific case.

This case report highlights two fundamental aspects of the approach to the patient with atrial fibrillation. The first is the need to assess the presence of structural atrial disease, and the second is the etiological investigation.

In persistent AF pathogenesis, atrial structural remodelling is essential and mainly involves fibrosis (23, 24). Therefore, assessing the extension and degree of atrial fibrosis is crucial in determining treatment options, predicting long-term evolution, and evaluating the substrate critical in the pathophysiology of atrial thrombogenesis.

The exposed clinical case also represents an example of deep etiological investigation, leading to identifying a rare genetic cause. This identification is essential in the genetic counselling of the patient and the broader contribution to the knowledge of the etiopathogenesis of atrial fibrillation.

In the upcoming years, it is expected to identify other AF-related genes in more extensive association studies, exome sequencing, and genome sequencing studies (25).

Not only the investigation of large populations will form the basis for the advancement of knowledge in this area, but we also believe that the reporting of clinical cases, such as the one we carry out in this article, also represents a contribution to advance in medical knowledge since a large part of the phenotype of specific genes is uncertain. Furthermore, retrospective information on other patients with the same genetic alterations is essential for prognosis.

## Data Availability

The datasets for this article are not publicly available due to concerns regarding participant/patient anonymity. Requests to access the datasets should be directed to the corresponding author.
