# Supplementary material for: Case report: Mutation in NPPA gene as a cause of fibrotic atrial myopathy
Source: Front Cardiovasc Med. 2023 Jun 8;10:1149717. doi: 10.3389/fcvm.2023.1149717 (PMC10285104; doi:10.3389/fcvm.2023.1149717)
Supplement: Supplementary file 1 [file Table1.docx]

SUPPLEMENTARY MATERIAL

The targeted panel for the inherited cardiac disease was analyzed:

A2ML1, AARS2, ABCC9, ACADVL, ACTA1, ACTC1, ACTN2, AGL, AKAP9, ALG10, ALMS1, ALPK3, ANK2, ANKRD1, ATP5E, BAG3, BRAF, CACNA1C, CALM1, CALR3, CASQ2, CAV3, CAVIN4, CBL, CDH2, CHRM2, COA5, CPT2, CRYAB, CSRP3, CTF1, CTNNA3, DES, DMD, DNAJC19, DOLK, DSC2, DSG2, DSG3, DSP, DTNA, ELAC2, EMD, EPG5, EYA4, FHL1, FHL2, FHOD3, FKRP, FKTN, FLNC, FOXRED1, FXN, GAA, GATA4, GATA6, GATAD1, GLA, GLB1, GPD1L, GUSB, HAMP, HCN4, HFE, HFE2, HRAS, IDH2, ILK, JPH2, JUP, KCND3, KCNE1, KCNE2, KCNE3, KCNH2, KCNJ2, KCNJ5, KCNQ1, KLF10, KRAS, LAMA4, LAMP2, LDB3, LMNA, LRRC10, MAP2K1, MAP2K2, MED12, MIB1, MRPL3, MTO1, MYBPC3, MYH6, MYH7, MYL2, MYL3, MYLK2, MYO6, MYOM1, MYOZ1, MYOZ2, MYPN, NEBL, NEXN, NF1, NKX2-5, NPPA, NRAS, OBSCN, PDLIM3, PKP2, PLEKHM2, PLN, PPA2, PRDM16, PRKAG2, PSEN1, PSEN2, PTPN11, RAB3GAP2, RAF1, RASA1, RBM20, RIT1, RPL3L, RRAS, RYR2, SCN1B, SCN3B, SCN4B, SCN5A, SCO2, SDHA, SGCA, SGCB, SGCD, SHOC2, SLC22A5, SLC25A3, SLC25A4, SLC40A1, SNTA1, SOS1, SOS2, SPRED1, SYNE1, SYNE2, TAZ, TCAP, TFR2, TGFB3, TJP1, TMEM43, TMEM70, TMPO, TNNC1, TNNI3, TNNI3K, TNNT2, TPM1, TRDN, TRIM63, TRPM4, TSFM, TTN, TTR, TXNRD2, VCL, XK.
